# Global Skin Cancer Burden From 1990 to 2023 and Projection to 2050

**DOI:** 10.1001/jamadermatol.2026.0964

**Published:** 2026-05-13

**Authors:** Youyou Zhou, Weiming Zhong, Xulei Liu, Jianglin Zhang

**Affiliations:** 1 Department of Dermatology, Shenzhen People’s Hospital, The First Affiliated Hospital, School of Medicine, Southern University of Science and Technology, The Second Clinical Medical College, Jinan University, Shenzhen, Guangdong, China; 2 Department of Neurosurgery, The Second People’s Hospital of Shenzhen, The First Affiliated Hospital of Shenzhen University, Shenzhen, Guangdong, China

## Abstract

This cross-sectional study uses the Global Burden of Disease database to summarize epidemiology, subgroup patterns, and projections for malignant melanoma, cutaneous squamous cell carcinoma, and basal cell carcinoma.

Malignant skin cancers impose an escalating and heterogeneous health burden worldwide.^1^ Using the Global Burden of Disease (GBD) 2023 database,^2^ we summarize these cancers’ epidemiology, subgroup patterns, and projections to 2050.

## Methods

We analyzed GBD 2023 estimates (1990-2023) for malignant melanoma, cutaneous squamous cell carcinoma, and basal cell carcinoma. Outcomes included prevalence and disability-adjusted life-years (DALYs; years of life lost due to premature death plus years lived with disability). Subgroup analyses were conducted by sex, age group, and Sociodemographic Index (SDI; range, 0-1), defined as the geometric mean of indices of fertility in those younger than 25 years, education among those aged 15 years and older, and lag-distributed income per capita. Projections to 2050 used a bayesian age-period-cohort (BAPC) model, a bayesian hierarchical framework that jointly estimates age, period, and cohort effects and provides uncertainty intervals. For basal cell carcinoma, we excluded the 2005 to 2009 surveillance-artifact period and fit projections using 2010 to 2023 data. Additional methods are provided in the eMethods in Supplement 1. This study was deemed to be not human participant research by Shenzhen People’s Hospital; therefore, institutional review board approval and informed consent were not required.

## Results

**Figure 1.**
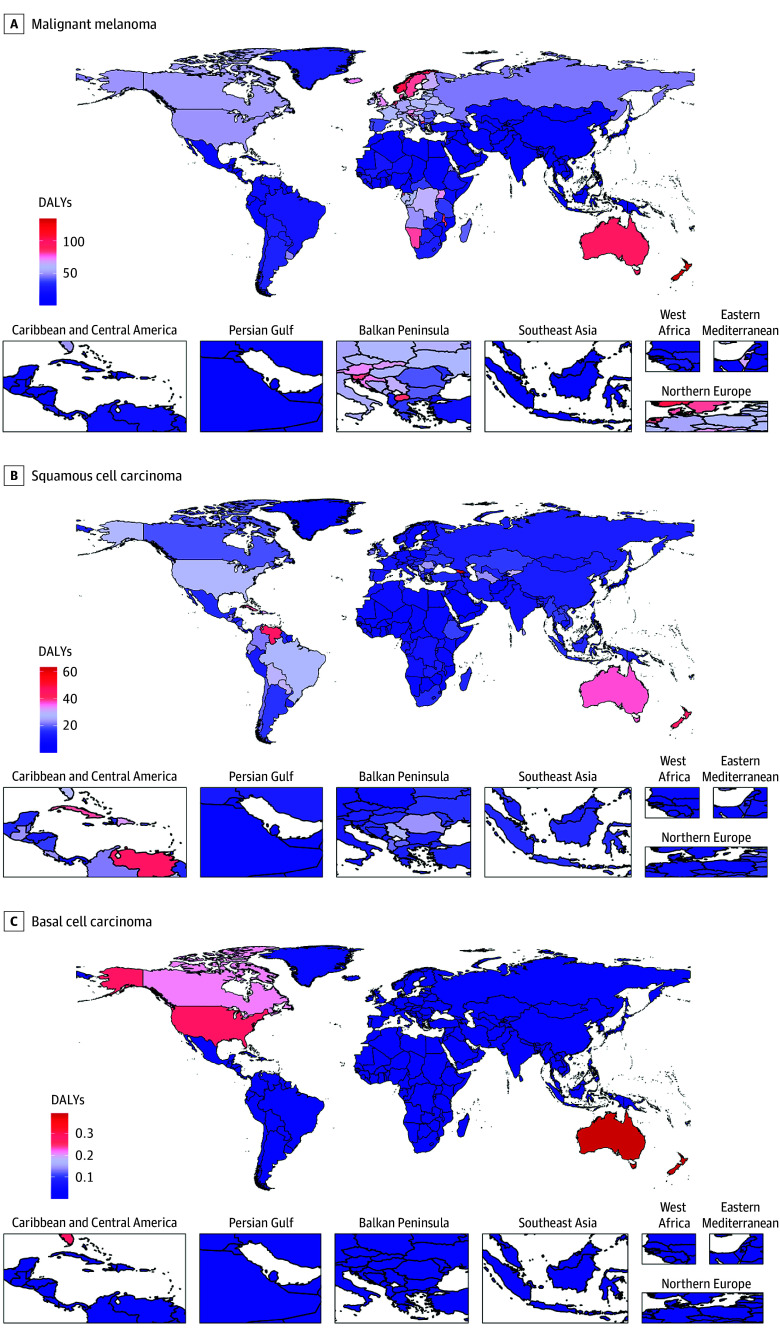
Heat Maps of Geographical Distribution of Global Skin Cancer Disability-Adjusted Life-Years (DALYs) in 2023 Geographical distribution of DALYs per 100 000 population caused by the 3 skin cancers. A, The DALYs for malignant melanoma are highest in Australia, New Zealand, and the Nordic countries. B, The DALYs for squamous cell carcinoma are concentrated in Australia, Brazil, and the Caribbean. C, The DALYs for basal cell carcinoma are highest in Australia and North America.

In 2023, skin cancers showed geographical variation, with DALYs concentrated in high-SDI regions (Figure 1). Prevalence exhibited a similar trend: melanoma was highest in Oceania (>300 cases per 100 000 population), while squamous cell carcinoma peaked in high-income Western countries, particularly the US (>200 cases per 100 000 population). Basal cell carcinoma was highest in Oceania, North America, and Northern Europe.

From 1990 to 2023, trends diverged across SDI levels. Low- and middle-SDI regions demonstrated consistent increases in incidence across the 3 cancers, with pronounced melanoma growth (258.8% in East Asia and 274.6% in Andean Latin America). The high-income Asia-Pacific region exhibited similar upward trends. In contrast, the high-income North America region showed a divergent pattern: melanoma incidence decreased (−10.5%) while squamous cell carcinoma and basal cell carcinoma increased (154.1% and 34.6%, respectively).

Melanoma DALYs declined globally, particularly in high-SDI regions (−36.1% in North America and −33.9% in Central Asia). Conversely, squamous cell carcinoma DALYs increased in low-SDI settings (93.2%). Basal cell carcinoma DALYs remained stable but increased notably in East Asia (45.1%) and the high-income Asia-Pacific region (39.6%).

By sex, male prevalence rates were consistently higher across the 3 cancers. For melanoma, prevalence in 2023 was 28.2 cases per 100 000 population in males and 25.6 cases per 100 000 population in females. Melanoma prevalence declined from 2010 to 2023 in both sexes (−17.8% in males and −14.1% in females). From 1990 to 2023, age-stratified analysis showed melanoma prevalence rising most steeply in older age groups (≥70 years), while younger cohorts (30-49 years) demonstrated declines. Decomposition analysis revealed that population growth was associated with the increasing case numbers for squamous cell carcinoma and basal cell carcinoma, while epidemiological changes contributed more to melanoma prevalence increases, especially in low-middle and middle-SDI regions.

**Figure 2.**
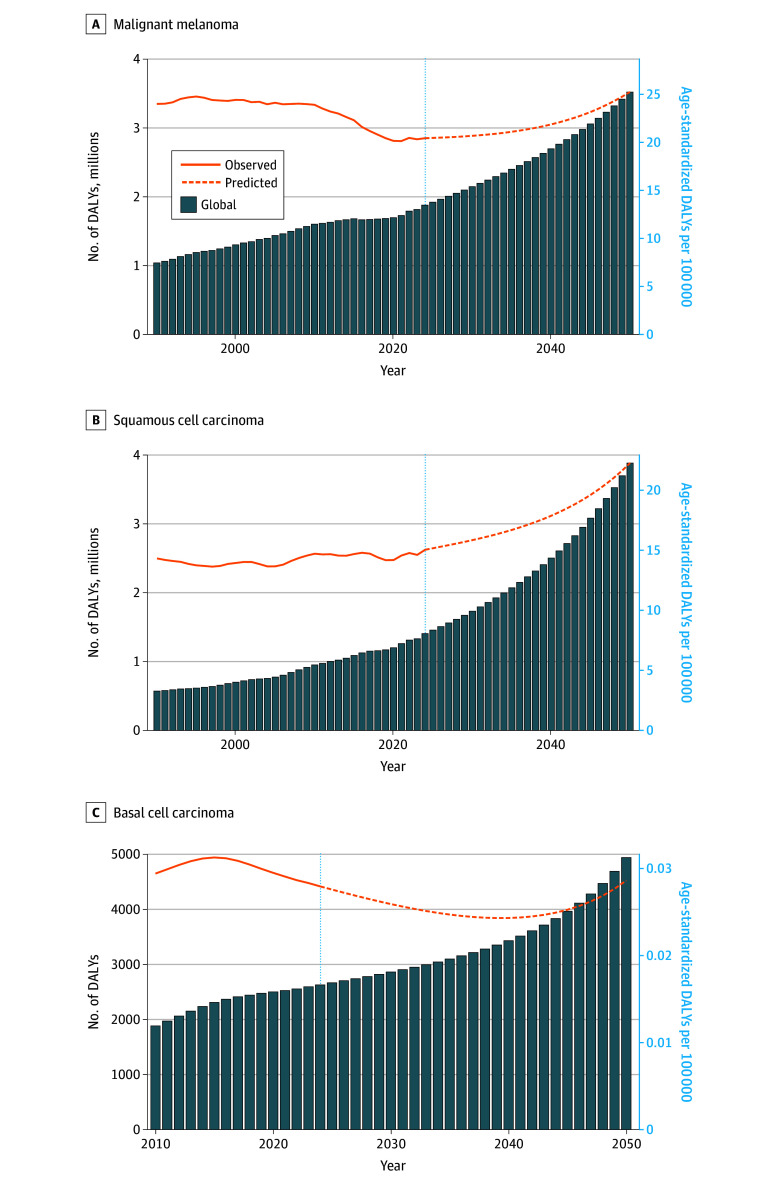
Bar Graphs Showing Prediction Analysis of Future Burden of Skin Cancers Based on Bayesian Age-Period-Cohort Model Bayesian age-period-cohort projections of combined-sex estimates of disability-adjusted life-years (DALYs) for malignant melanoma (A; 1990-2050), cutaneous squamous cell carcinoma (B; 1990-2050), and basal cell carcinoma (C; 2010-2050). For basal cell carcinoma, model fitting excluded the 2005 to 2009 period due to a transient surveillance artifact. Solid lines indicate observed data (1990-2023 for panels A and B, and 2010-2023 for panel C), with dashed lines indicating predicted values from 2024 to 2050. The vertical dotted blue line marks the transition from observed to predicted data. Panel C displays global data only due to numerical instability in regional long-term extrapolation.

Bayesian age-period-cohort projections indicate continued global burden increases through 2050, with melanoma DALYs rising from approximately 2 million in 2025 to more than 3.3 million in 2050, squamous cell carcinoma DALYs increasing from 1.2 million in 2025 to 4.0 million in 2050, and basal cell carcinoma accounting for the highest total DALYs, approaching 5.0 million in 2050. Low- and middle-SDI regions are projected to experience steep growth trajectories across all 3 cancers (Figure 2).

## Discussion

Findings of this cross-sectional study indicate distinct regional patterns. Low- and middle-SDI settings demonstrated increasing trends across all 3 cancers, while high-SDI settings showed heterogeneous patterns with melanoma burden stabilizing in some regions and continued challenges in basal cell carcinoma management.^3^ These patterns align with the United States Cancer Statistics (1999-2021 melanoma age-adjusted incidence, 15.1-23.0 cases per 100 000 population)^4^ and Australian cancer surveillance showing declining melanoma rates among individuals younger than 40 years alongside rising keratinocyte carcinoma burden.^5^ However, this GBD-based global analysis captures the rising burden in low- and middle-SDI regions—populations underrepresented in established high-income country registries. These differences may stem from differences in health care access and screening infrastructure across SDI levels. High-SDI regions benefit from well-established dermatological surveillance systems, routine skin examinations, and public awareness campaigns, facilitating earlier detection, whereas low- and middle-SDI settings often face limitations in specialized health care resources and population-based screening programs, potentially leading to underdiagnosis in early stages and contributing to the observed variations in incidence and prevalence patterns.

GBD estimates may underestimate burden in low-SDI settings due to incomplete reporting and limited health care infrastructure. Projections may be affected by public health policies and health care resource allocation.

## Notes

Supplement 1.
eMethods

Supplement 2.
Data Sharing Statement
