# Supplementary material for: Global Skin Cancer Burden From 1990 to 2023 and Projection to 2050
Source: JAMA Dermatol. 2026 May 13;162(7):745–8. doi: 10.1001/jamadermatol.2026.0964 (PMC13173422; doi:10.1001/jamadermatol.2026.0964)
Supplement: Supplement 1. — eMethods [file jamadermatol-e260964-s001.pdf]

Supplemental Online Content

Zhou Y, Zhong W, Liu X, Zhang J. Global skin cancer burden from 1990 to 2023 and projection to 2050. *JAMA Dermatol*. Published online May 13, 2026. doi:10.1001/jamadermatol.2026.0964

eMethods

This supplemental material has been provided by the authors to give readers additional information about their work.

© 2026 Zhou Y et al. *JAMA Dermatol*.

eMethods

Data source

GBD 2023 estimates the incidence, prevalence, mortality, years lived with disability (YLDs), years of life lost (YLLs), and disability-adjusted life years (DALYs) of 369 diseases and injuries worldwide, covering 23 age groups and 204 countries and territories, which are divided into 21 geographical regions since 1990. All indicators are expressed as numbers, rates, and percentages, which can be selected by sex, age, and region according to research needs. In this study, we used the data of malignant cutaneous melanoma, non-melanoma skin cancer (squamous cell carcinoma), and non-melanoma skin cancer (basal cell carcinoma) from 1990 to 2023 from the GBD 2023 database, and included data for males, females, and all sexes.

SDI

The Sociodemographic Index (SDI) quantifies the level of development of a country or region through fertility rate, education level, and per capita income data. The SDI ranges from 0 to 1, with higher values indicating higher levels of socioeconomic development. The SDI is known to be associated with disease incidence and morbidity. In this study, we divided countries and regions into five SDI categories (low, low-moderate, medium, medium-high, and high) to examine the relationship between the burden of skin melanoma, non-melanoma skin cancer (squamous cell carcinoma and basal cell carcinoma) and socioeconomic development.

ASR

In order to make meaningful comparisons of various rates between different populations around the world, the same standard population was used in the GBD study, and age standardization was used to adjust the rates of different risk factors in a country. Age-standardized rate (ASR) is a commonly used indicator in epidemiology. When the age structure of multiple comparison groups is different, direct comparison of crude rates will lead to bias, because it cannot show whether the high incidence rate

© 2026 Zhou Y et al. *JAMA Dermatol.*

in a certain area is due to differences in age structure, so it is usually necessary to compare after standardization. Therefore, age standardization reflects the level that is not affected by age factors, does not represent an absolute rate, but facilitates the comparison of data from different regions or different periods. The age-standardized rate per 100,000 population is equal to the sum of the product of the specific age of each age group and the number of cases in the same age group in the selected reference standard population, divided by the sum of the standard population weights. The calculation of age-standardized rate uses the GBD world population as the reference standard.

Decomposition analysis

The purpose of decomposition analysis is to quantitatively analyze the driving factors that cause changes in a certain indicator, that is, to obtain the effect of the driving factor. For the burden of disease, the effect obtained by decomposition analysis refers to the impact of a change in a certain factor on the total change when the year changes and other driving factors remain unchanged. For example, the global burden of disease caused by diseases has increased rapidly in the past 30 years. We want to know how much of the increased burden of disease in the past 30 years comes from population growth, how much comes from population aging, and how much comes from epidemiological changes. Decomposition analysis can give a static and intuitive display.

Prediction model

The BAPC model is developed on the basis of the APC model. Its theoretical basis is derived from the generalized linear model (GLM). By introducing the Bayesian framework and prior information, the model has better stability and explanatory power in small samples or data with large uncertainty. In this study, we used the BAPC model to predict the DALYs of skin melanoma and non-melanoma skin cancer (squamous cell carcinoma and basal cell carcinoma), evaluate their changing trends in the next few decades, and analyze the possible disease burden risks. Because BCC

© 2026 Zhou Y et al. *JAMA Dermatol.*

data contains a transient surveillance artifact during 2005-2009, the BAPC model misinterpreted this period as accelerated disease growth. Our joinpoint regression analysis reveals that BCC exhibited an extreme +18.11% annual percent change during 2005-2009—more than 10-fold higher than any melanoma or SCC rate change. This surge immediately moderated to +3.04% (2009-2014) and reversed to -1.40% (2014-2023), confirming it reflects improving surveillance systems rather than biological disease trends. In contrast, melanoma and SCC showed stable patterns with all annual percent changes between -2% and +2%, indicating reliable surveillance suitable for projection. Therefore, we excluded data from 2005–2009 and conducted projections using only the 2010–2023 BCC data.

Statistical analysis

The prevalence and disability-adjusted life years (DALYs) were predicted per 100,000 population. All analysis and graphical representation processes were performed using the statistical computing software R (version 4.3.2).

© 2026 Zhou Y et al. *JAMA Dermatol.*
