# Supplementary material for: Global Skin Cancer Burden From 1990 to 2023 and Projection to 2050
Source: JAMA Dermatol. 2026 May 13;162(7):745–8. doi: 10.1001/jamadermatol.2026.0964 (PMC13173422; doi:10.1001/jamadermatol.2026.0964)
Supplement: Supplement 2. — Data Sharing Statement [file jamadermatol-e260964-s002.pdf]

Data Sharing Statement

Zhou. Global Skin Cancer Burden From 1990 to 2023 and Projection to 2050. *JAMA Dermatol*. Published May 13, 2026. doi:10.1001/jamadermatol.2026.0964

Data
Data available: Yes
Data types: Deidentified participant data
How to access data: Statistical code and raw data used in this study are available at https://pan.baidu.com/s/1XTa1ju0uUPowNSnt0hTXVQ. If there is a reasonable need, further contact can be made with the corresponding author.
When available: With publication

Supporting Documents
Document types: Statistical/analytic code
How to access documents: Statistical code and raw data used in this study are available at https://pan.baidu.com/s/1XTa1ju0uUPowNSnt0hTXVQ. If there is a reasonable need, further contact can be made with the corresponding author.
When available: With publication

Additional Information
Who can access the data: anyone requesting the data with a reasonable need
Types of analyses: For epidemiological analysis of skin tumor prevalence and disability-adjusted life years (DALYs).
Mechanisms of data availability: The data will be made available through a public repository (https://pan.baidu.com/s/1XTa1ju0uUPowNSnt0hTXVQ) after approval of a proposal or with a signed data access agreement if required.
